# Mammographic breast density and its association with urinary estrogens and the fecal microbiota in postmenopausal women

**DOI:** 10.1371/journal.pone.0216114

**Published:** 2019-05-08

**Authors:** Gieira S. Jones, Heather Spencer Feigelson, Roni T. Falk, Xing Hua, Jacques Ravel, Guoqin Yu, Roberto Flores, Mitchell H. Gail, Jianxin Shi, Xia Xu, James J. Goedert

**Affiliations:** 1 Department of Epidemiology, University of North Carolina Chapel Hill, Chapel Hill, North Carolina, United States of America; 2 Institute for Health Research, Kaiser Permanente Colorado, Denver, Colorado, United States of America; 3 Division of Cancer Epidemiology and Genetics, National Cancer Institute, Bethesda, Maryland, United States of America; 4 Institute for Genome Sciences, University of Maryland School of Medicine, Baltimore, Maryland, United States of America; 5 Division of Cancer Prevention, National Cancer Institute, Bethesda, Maryland, United States of America; 6 Cancer Research Technology Program, Leidos Biomedical Research Incorporated, Frederick National Laboratory for Cancer Research, Frederick, Maryland, United States of America; Medical University of South Carolina, UNITED STATES

## Abstract

**Background:**

Breast density, as estimated by mammography, is a strong risk factor for breast cancer in pre- and postmenopausal women, but the determinants of breast density have not yet been established. The aim of this study was to assess if urinary estrogens or gut microbiota alterations are associated with mammographic density in postmenopausal women.

**Methods:**

Among 54 cancer-free, postmenopausal controls in the Breast and Colon Health study, we classified low- versus high-density women with Breast Imaging Reporting and Data System (BI-RADS, 5^th^ edition) mammographic screening data, then assessed associations with urinary estrogens and estrogen metabolites (determined by liquid chromatography/tandem mass spectrometry), and fecal microbiota alpha and beta diversity (using Illumina sequencing of 16S rRNA amplicons).

**Results:**

Multiple logistic regression revealed no significant association between breast density and fecal microbiota metrics (PD_tree P-value = 0.82; un-weighted and weighted UniFrac P = 0.92 and 0.83, respectively, both by MiRKAT). In contrast, total urinary estrogens (and all 15 estrogens/estrogen metabolites) were strongly and inversely associated with breast density (P = 0.01) after adjustment for age and body mass index.

**Conclusion:**

Mammographic density was not associated with the gut microbiota, but it was inversely associated with urinary estrogen levels.

**Impact:**

The finding of an inverse association between urinary estrogens and breast density in cancer-free women adds to the growing breast cancer literature on understanding the relationship between endogenous estrogens and mammographic density.

## Introduction

Mammographic density is a metric used to describe the amount of adipose tissue relative to the amount of connective, lobular, and ductal tissue in the breast. High mammographic density is a strong risk factor for breast cancer, but its determinants are not well established [1–5]. To further complicate the matter, how mammographic density increases breast cancer risk is unclear [2]. Several epidemiological studies have implicated endogenous estrogen levels as a possible contributor to the breast cancer–density association [6–20]. Since estrogen functions as a mitogen that regulates breast tissue growth and development, it is biologically plausible that estrogen may mediate the relationship between mammographic density and breast cancer [21]. However, associations of endogenous estrogen levels with mammographic density have been inconsistent, including null, positive, and inverse correlations [6,7,16–20,8–15].

The intestinal microbiota plays an important role in human health and disease by modulating hormone homeostasis as well as digestion, metabolism, and immune conditioning [22,23]. Research on host-microbiota interactions and how they relate to disease has revealed that the microbiota may be associated with breast cancer [24,25]. The gut microbiota affects estrogen levels through enterohepatic circulation [26,27], and one study has assessed the relationship of breast cancer with both the gut microbiota and estrogen levels [24]. Specifically, we recently reported that postmenopausal women with breast cancer had a less diverse gut microbiota and an altered microbial community compared to matched control women from the same population. To date, no study has evaluated whether breast density is influenced by the microbiome. We had the opportunity to evaluate this in postmenopausal women identified as healthy controls for a pilot case control breast cancer study investigating the gut microbiome. The aim of the current study was to determine if mammographic density in cancer-free women was associated with gut microbiota metrics, urinary estrogen levels, or both.

## Materials and methods

### Selection and recruitment of study participants

Following review and approval of the protocol and procedures by institutional review boards at Kaiser Permanente Colorado (KPCO) and the National Cancer Institute, 54 postmenopausal women aged 50–74 years old who had recently received a normal screening mammogram were recruited. These women served as controls for a previous study that has been described in detail elsewhere [28,29]. Briefly, the virtual data warehouse (VDW) at KPCO was used to identify eligible women (approximately n = 40,000). The VDW is a standardized database of inpatient and outpatient diagnoses, procedures, laboratory results, and medications derived from the electronic medical record. Using the VDW, we excluded women who had prescribed medications or conditions that could strongly impact the normal gut microbial population or circulating hormone levels including the following: any history of prior cancer (other than non-melanoma skin cancer), inflammatory bowel disease or diverticulitis, gastric banding or by-pass surgery; history of other gastric or intestinal surgery (such as appendicitis) within the previous 6 months, any progesterone or estrogen prescription within the previous 12 months, and antibiotic prescription within the previous 6 months.

Women who fit the study criteria were randomly sampled and invited to enroll in the study. Participants received a phone call explaining the study, obtaining verbal consent, and verifying eligibility, including menopausal status. Consented women then received a specimen collection kit and questionnaire by mail.

### Questionnaires, demographic and clinical data

The first page of the self-administered questionnaire included a prominent, explicit statement indicating that, by completing and submitting the questionnaire, the participant was providing consent to participate in the study. This document, as well as this consent and all the other procedures, were reviewed and approved by the Kaiser Permanente Colorado and National Cancer Institute Institutional Review Boards (IRB). Consent was documented upon receipt of the document. The self-administered questionnaire also included demographic, smoking, alcohol consumption, family history of cancer, education, physical activity, body habitus, previous surgery, reproductive and menstrual history, and recent dietary data (S1 File).

Electronic medical records were reviewed to obtain additional clinical and demographic data including length of KPCO enrollment, BMI, Breast Imaging Reporting and Data System (BI-RADS) category, number of prior mammograms, radiology code for recent mammograms, history of fecal occult blood testing, and clinical chemistry and hematology parameters within the previous year.

### Specimen collection

The specimen kit contained written and illustrated instructions, stool collecting pouch, and a zipped “lunch box” containing a foam insert for holding four Sarstedt tubes for fecal collection and a 120 ml screw top container for urine collection. All specimen collection tubes were pre-coded with barcodes without personal identifiers. To collect fecal specimens, participants attached the stool catching pouch to the toilet seat and after defecating normally used the scoop on each of the Sarstedt tubes to obtain four aliquots from one stool. Of the four Sarstedt tubes, two were pre-loaded with 5 ml RNA later and two with 5 ml of sterile phosphate buffered saline. The participants also collected contemporaneous urine in the screw top container (without preservative). After procuring all of the specimens and storing them in their respective slots within the specimen kit, the participant stored the entire sealed kit in her home freezer for pick up and transport on dry ice by a KPCO staff member. The specimens were stored frozen at -20°C in the KPCO laboratory and subsequently shipped in batches to the NCI repository where they were stored at -80°C until use.

### Urine estrogens and estrogens metabolites

Stable isotope dilution liquid chromatography/tandem mass spectrometry (LC-MS/MS) was used to measure urinary concentrations of the parent estrogens (estrone and estradiol) and 13 estrogen metabolites including 2-hydroxylated estrogen metabolites (2-hydroxyestrone, 2-methoxyestrone, 2-hydroxyestradiol, 2-methoxyestradiol, and 2-hydroxyestrone-3-methyl ether); 4-hydroxylated estrogen metabolites (4-hydroxyestrone, 4-methoxyestrone, and 4-methoxyestradiol); and 16-hydroxylated estrogen metabolites (16α-hydroxyestrone, estriol, 17-epiestriol, 16-ketoestradiol, and 16-epiestriol). Details of the method, including sample preparation and assay conditions, have been published previously [30]. For this study, updated LC-MS/MS instruments and additional stable isotope labeled estrogens and estrogen metabolites were employed. Briefly, LC-MS/MS analysis was performed using a Thermo TSQ Quantum Ultra triple quadrupole mass spectrometer (Thermo Scientific, San Jose, CA) coupled with a Prominence UFLC system (Shimadzu Scientific Instruments, Columbia, MD). Both LC and MS were controlled by Xcalibur software (Thermo Scientific). Twelve stable isotopically labeled estrogens and estrogen metabolites were used to account for losses during sample preparation and assays, which included deuterated estriol, (C/D/N Isotopes, Inc., Pointe-Claire, Quebec, Canada); deuterated 16-epiestriol (Medical Isotopes, Inc., Pelham, NH); and 13C-labeled estrone, estradiol, 2-hydroxyestrone, 2-methoxyestrone, 2-hydroxyestradiol, 2-methoxyestradiol, 2-hydroxyestrone-3-methyl ether, 4-hydroxyestrone, 4-methoxyestrone, and 4-methoxyestradiol (Cambridge Isotope Laboratories, Andover, MA). To standardize the values, creatinine was measured in the urine specimens by the Clinical Chemistry laboratory at the NIH Clinical Center.

This analysis includes parent estrogens (estrone plus estradiol), estrogen metabolites grouped into three major hydroxylation pathways (2-, 4- and 16-hydroxylation); total metabolites (parent estrogens excluded); total estrogens (sum of estrone, estradiol and all metabolites); the ratio of total metabolites to parent estrogen (overall, and separately for each hydroxylation pathway); and the ratio of 2-pathway to 16-pathway metabolites. In quality-control replicate samples interspersed with the test samples, laboratory coefficients of variation were <7% and intraclass correlation coefficients >87% for metabolite concentrations.

### Fecal DNA extraction and 16S rRNA amplification, sequencing, and analysis

DNA isolation and purification were performed by the Institute of Genome Sciences, University of Maryland School of Medicine as described previously [24,31]. As previously described in detail, approximately 469 bp of the 16S rRNA gene V3-V4 hypervariable region of the fecal DNA was amplified with primers that included a linker sequence (suitable for the MiSeq 300PE Illumina sequencer, San Diego CA), a 12 bp index sequence, a heterogeneity spacer (to minimize bias with low-diversity amplicons), and 16S rRNA universal primers 319F/806R. DNA products were quantified by Qubit Fluorometer (Life Technologies, Grand Island NY). The amplicons were sequenced in a single pool in one run on the MiSeq instrument using the 300PE protocol, generating approximately 2.22 Gb of data. The raw sequences were processed to concatenate forward and reverse reads and to sort and match paired end sequences and barcodes using the published pipeline [31]. The processed reads were clustered and the operational taxonomic units (OTUs) were assigned to taxa by matching to the Ribosomal Data Project naïve Bayesian classifier [32]. Richness (number of observed species) and alpha diversity metrics (Chao1, Shannon index, and Phylogenetic Diversity whole-tree) were calculated after rarifying to 12,599 reads using the Quantitative Insights Into Microbial Ecology (QIIME) pipeline [33]. Based on 13 replicate specimens from the MicroBiome Quality Control project that were included at all steps [34], coefficient of variation (CV) of Shannon index was 1.02%. Six women were excluded from the microbiome analysis due to unsatisfactory fecal microbiota data.

### Mammographic density

The principal dependent variable, mammographic density, employed categories from the Breast Imaging Reporting and Data System (BI-RADS, 5^th^ edition), which were combined into low density [almost entirely fatty tissue, scattered fibroglandular tissue (categories A and B)] versus high density [heterogeneously dense and extremely dense (categories C and D)] [35]. When this classification was used by five radiologists for 1000 patients, including 100 re-read one month later, inter-reader agreement was nearly perfect (overall weighted kappa 0.88) [36]. The four original categories (A, B, C, and D) were considered in sensitivity analyses.

### Statistical analysis

Body mass index (BMI) was calculated as weight/height-squared (kg/m^2^). Mean differences in symmetrically distributed continuous independent variables such as age were assessed by Student’s T-Test. Non–Gaussian distributed continuous variables including age at menopause, age at menarche, years since menopause, 5-year and lifetime risk of breast cancer (Gail risk score), and principal component scores of weighted and un-weighted UniFrac metrics [33] were assessed using Wilcoxon rank sum test. To assess differences in covariate distribution between low and high mammographic density women chi–square analysis was used for categorical variables [race, college attendance, BMI categories, smoking status (ever/never), current alcohol use, history of family cancer, previous use of hormone replacement therapy, at least one live birth, and moderate/vigorous physical activity]. The relationship between natural log levels of estrogens and estrogen metabolites (continuous variable) and mammographic density was assessed using unconditional logistic regression models with adjustments for age and BMI. In this study all microbiota metrics are comprised solely of the bacterial and archaeal population, and does not include protozoa, fungi or viruses. Associations of microbiota richness and alpha diversity with natural log levels of estrogens and estrogen metabolites (measured as pmol per mg creatinine) were tested by Spearman rank-order correlation and multiple linear regressions. Associations of richness and alpha diversity with mammographic density were tested by unconditional logistic regression. BMI and age, which are known negative confounders of mammographic density [21] and also may be associated with the gut microbiota [26], were added as continuous covariates in all logistic regression models. Wilcoxon rank sum tests were used to analyze the association of mammographic density and genus level taxa; nominal p-values were reported, without adjustment for multiple comparisons, because this small study can only give exploratory findings at the genus level. MiRKAT regression analysis [37] was used to test for associations of mammographic density with beta diversity, specifically with un-weighted and weighted UniFrac distance matrices as kernels in the model. Significance was assessed by 10,000 permutations. All statistical tests were two-sided, and statistical significance was defined as P < 0.05.

## Results

Twenty-three women were classified as having low mammographic density, and 31 were classified has having high mammographic density. Participants were predominantly non-Hispanic white (86%), and nearly all (96%) had attended college, which did not differ by mammographic density status. Likewise, women with low versus high mammographic density were similar with regard to age, histories of cigarette smoking, alcohol use, vigorous/moderate physical activity, prior HRT use, breast cancer in a first-degree family member, menstruation history and parity (Table 1). Low mammographic density women had significantly higher mean BMI (P = 0.01), with 48% of low-density women being categorized as obese compared to 13% of high-density women.

**Table 1:** Demographics of 54 postmenopausal women without breast cancer, by mammographic density*.

	Low Density (n = 23)	High Density (n = 31)	p-value*
Mean Age(SD)	61.17 (3.7)	61.8 (3.6)	0.48
White Non—Hispanic %	91%	81%	0.35
Attended College	95%	96%	0.88
Mean BMI	29.64 (4.9)	26.18 (5.73)	0.01
Normal	17%	52%	0.01
Overweight	35%	35%	
Obese	48%	13%	
Ever Smoked 100 Cigarettes	54%	41%	0.36
Currently Drink Alcohol	77%	77%	0.99
Vigorous Physical Activity			
No vigorous physical activity	27%	29%	0.38
< 2 hours of vigorous activity/week	41%	55%	
> 2 hours of vigorous activity /week	32%	16%	
Moderate Physical Activity			
No moderate physical activity	14%	6%	0.65
< 2 hours of moderate activity/week	36%	36%	
> 2 hours of moderate activity /week	50%	58%	
Gail score			
Median 5- year risk	1.8 (0.17)	1.7 (0.19)	0.55
Median lifetime risk	8.7 (0.69)	7.9 (0.84)	0.33
History of Hormone Replacement Therapy	36%	56%	0.18
History of Family Cancer	21%	16%	
*Reproductive information*	*N = 22*	*N = 27*	
Median age at menarche, SE	13 (0.32)	13 (0.31)	0.31
Median age at menopause SE	52 (1.4)	50 (1.8)	0.12
Median years since menopause, SE	9 (1.6)	11 (1.9)	0.15
*Reported parity Information*	*N = 19*	*N = 23*	
At least one live birth	74%	87%	0.27

*Symmetrically distributed continuous variables such as age were assessed by Student’s T-Test. Non–symmetrically distributed continuous variables including age at menopause, age at menarche, years since menopause, 5-year and lifetime risk of breast cancer (Gail risk score) were assessed using Wilcoxon rank sum test. Categorical variables were analyzed using chi–square analysis (race, college attendance, body mass index categories, ever smoked, current alcohol use, history of family cancer, previous use of hormone replacement therapy, at least one live birth, and moderate/vigorous physical activity).

**Fig 1:**
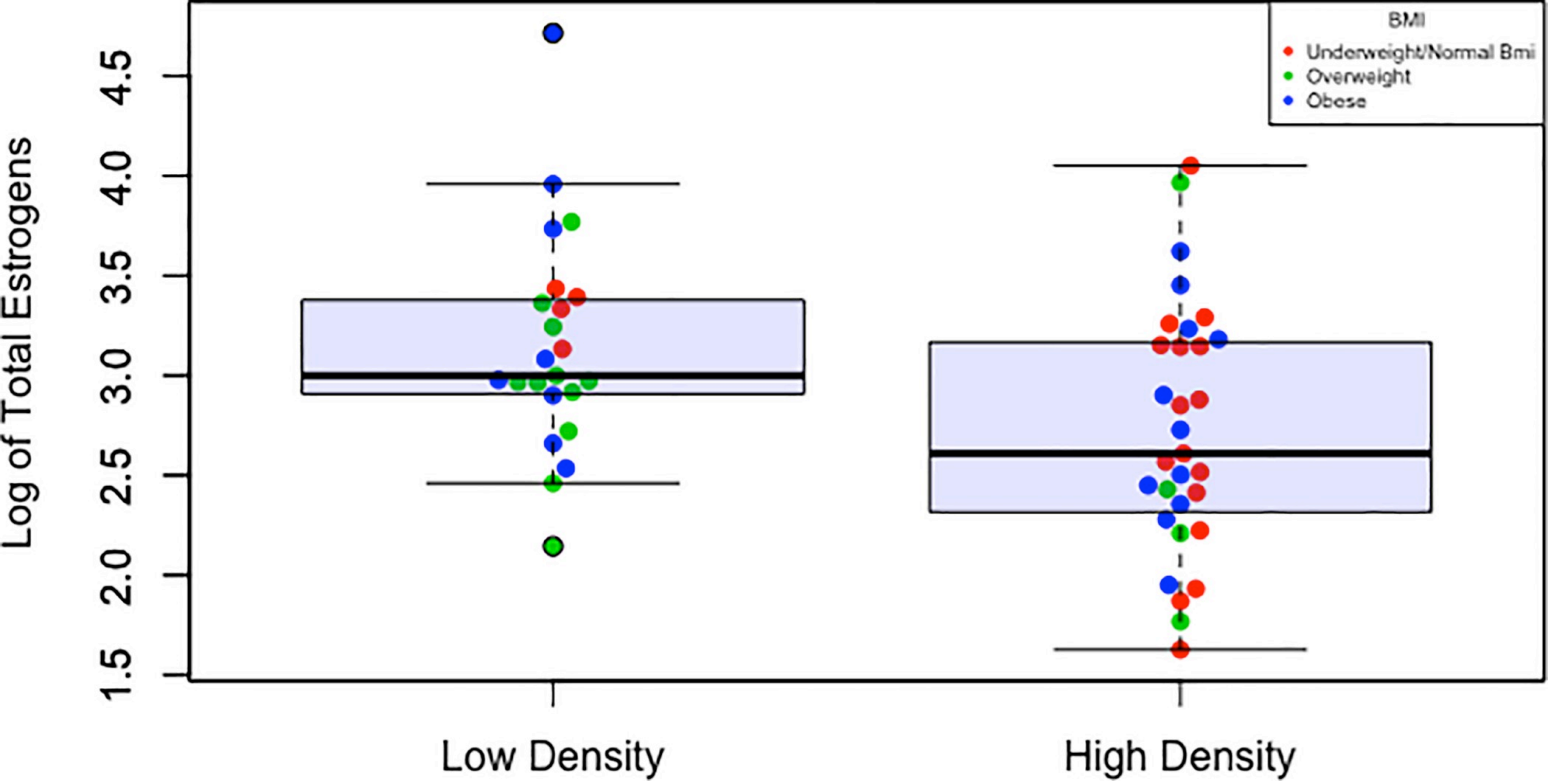
High mammographic density (MD) associated with both lower body mass index (Kg/M^2^, P = 0.04) and lower total estrogen level (pmol/mg creatinine, P = 0.02) among postmenopausal women. The logistic regression model for MD included age as a covariate.

Women with low mammographic density had significantly higher levels of all urinary estrogens and estrogen metabolites (adjusted for BMI, geometric mean total level 23.81 pmol/mg creatinine versus 14.88 pmol/mg creatinine in high-density women, P = 0.01, Table 2). Likewise, estrone (E1), estradiol (E2), and the 13 estrogen metabolites grouped by metabolic pathway were all higher in low-density women than in high-density women, with or without adjustment for BMI (all P≤0.05). In contrast, estrogen parent: metabolite and metabolite-pathway ratios did not differ between low and high mammographic density women. As shown in Fig 1, high mammographic density (MD) was associated with both lower BMI and lower estrogen level (P = 0.04 and 0.02, respectively). Overall, BMI was not significantly correlated with total estrogen level (Spearman R = -0.02, P = 0.88), but in women whose mammograms were characterized as low density (entirely fatty or scattered fibroglandular tissue) total estrogens tended to be higher in those with lower BMI (β = -0.029, Fig 1). A test of interaction (BMI*estrogen level for density) was not significant (P = 0.77). Splitting out the few mammograms classified as “Almost entirely fatty” (N = 6) or “Extremely dense” (N = 5) from low-density and high-density, respectively, did not affect the observed associations (S1 Table).

**Table 2:** Estrogen and estrogen metabolite levels by mammographic density category.

	Low Density		High Density		
	Adjusted Geometric Mean* (N = 23)	95%CI	Adjusted Geometric Mean* (N = 31)	95% CI	P-value
**Total Estrogen + EM**	23.81	18.92–30.27	14.88	11.94–18.92	0.01
**Estrone (E1)**	5.87	4.48–7.77	3.86	3.03–4.90	0.04
**Estradiol (E2)**	1.43	1.08–1.88	0.96	0.76–1.22	0.05
**Parent Estrogens (E1+E2)**	7.39	5.64–9.78	4.90	3.86–6.17	0.03
**Estrogen Metabolites (EM)**	15.96	12.55–20.29	9.87	7.77–12.43	0.02
**16-Pathway**	8.41	6.55–10.91	5.05	3.97–6.42	0.01
**2-Pathway**	6.82	5.42–8.58	4.35	3.46–5.58	0.02
**4-Pathway**	0.59	0.48–0.75	0.39	0.31–0.49	0.02
***EM Ratios***					
**EM/Parent Estrogens**	2.58	1.49–3.68	2.15	1.88–2.42	0.53
**16-Pathway/Parent**	1.49	0.61–2.38	1.11	0.96–1.27	0.52
**2-Pathway/Parent**	1.00	0.79–1.21	0.95	0.84–1.07	0.79
**4-Pathway/Parent**	0.09	0.07–0.11	0.08	0.07–0.10	0.87
**2-Pathway/16-Pathway**	0.83	0.75–0.91	0.88	0.82–0.94	0.34
**4-Pathway/Parent**	0.09	0.07–0.11	0.08	0.07–0.10	0.87
**2-Pathway/16-Pathway**	0.83	0.75–0.91	0.88	0.82–0.94	0.34

*Geometric means were adjusted for BMI as a continuous variable. Unconditional logistic regression adjusted for BMI and age.

**Fig 2:**
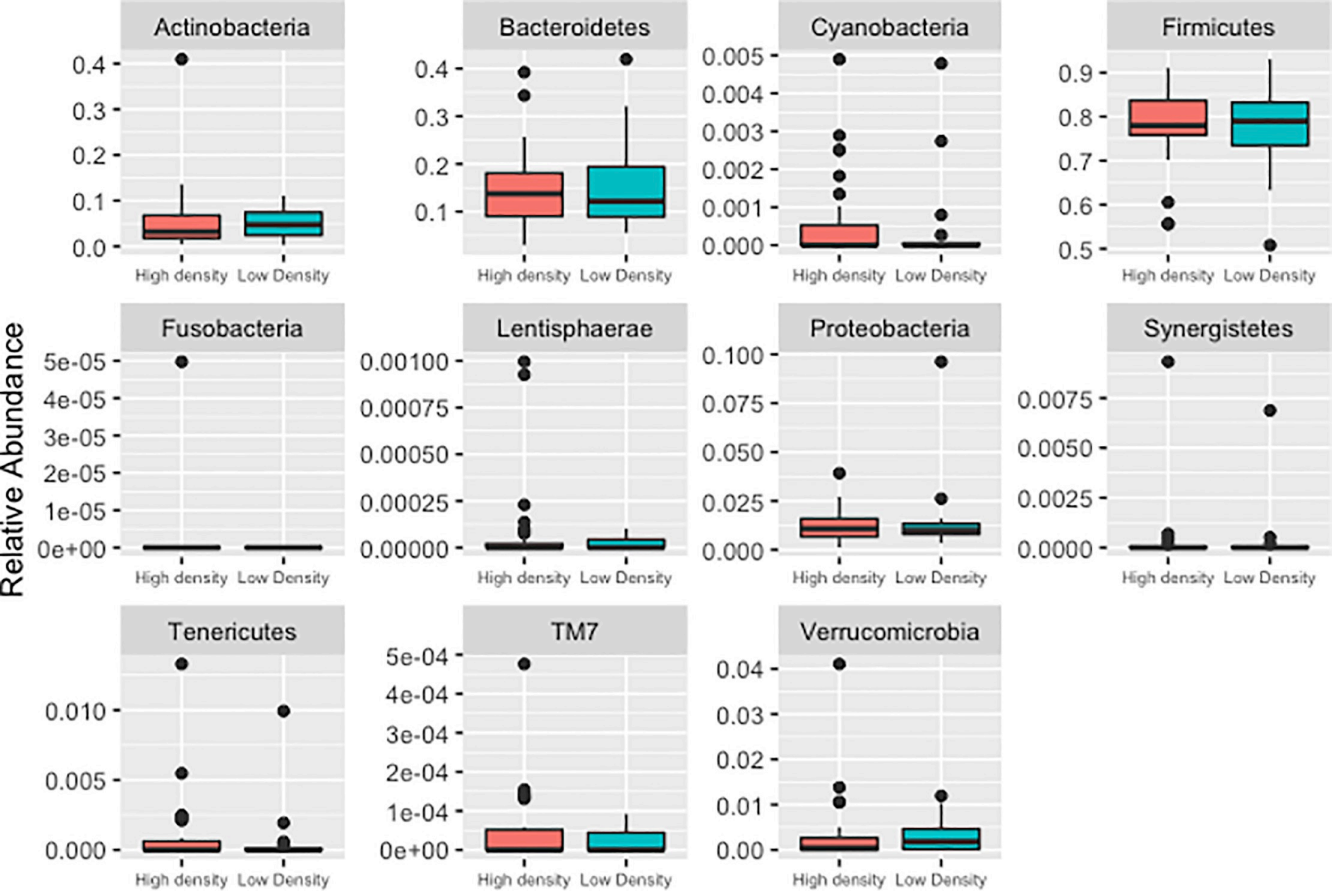
Box plot comparisons of microbiota relative abundance at the Phylum level among low and high mammographic density postmenopausal women.

**Fig 3:**
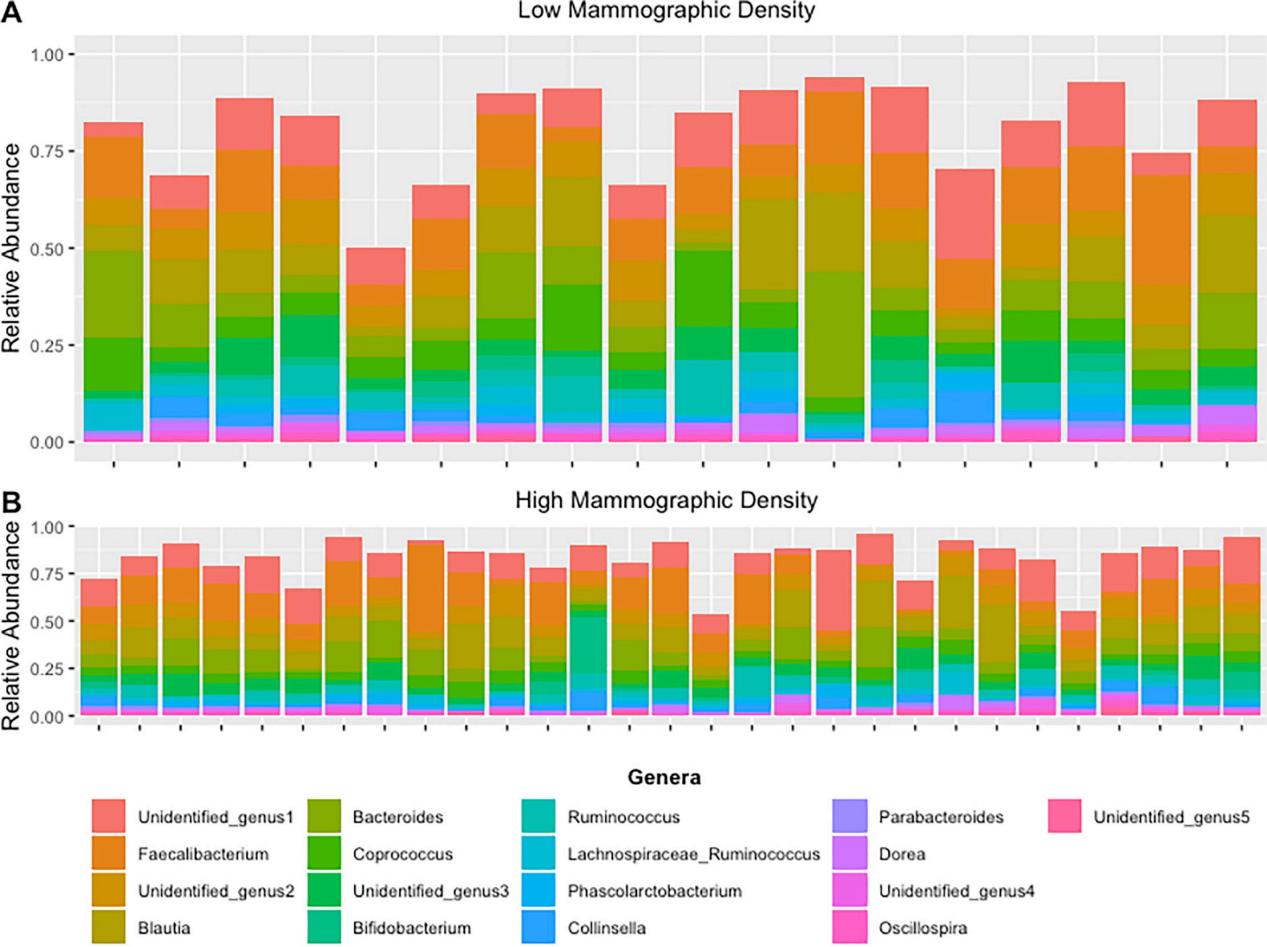
Stacked Bar Chart of the 90^th^ Percentile of most abundant microbiota at the Genus level among high and low mammographic density postmenopausal women.

**Fig 4:**
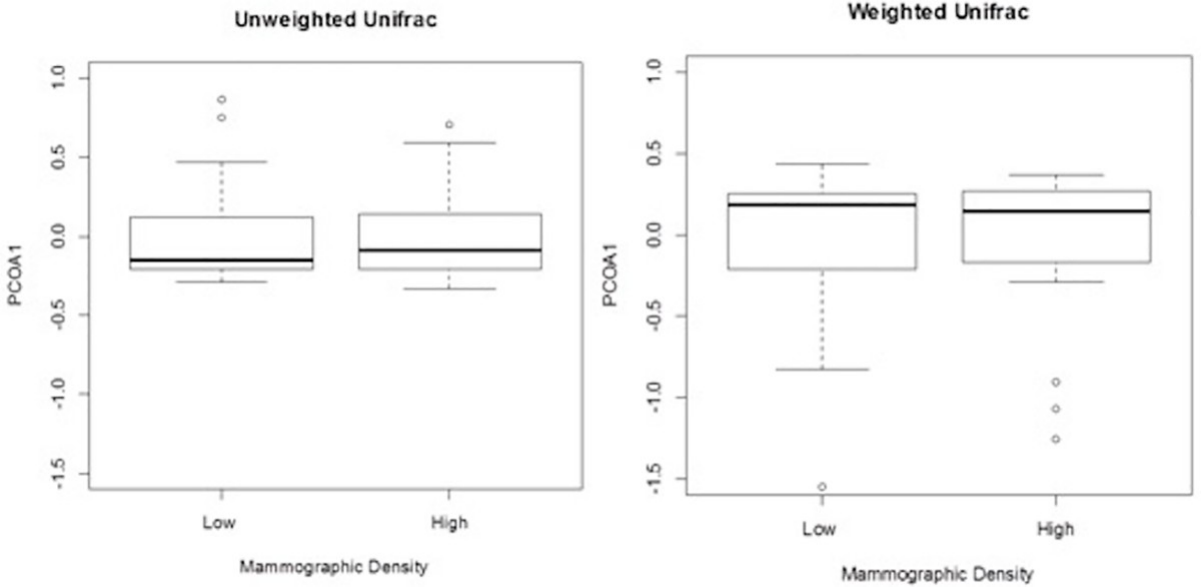
Box plot comparisons of beta diversity between low and high mammographic density postmenopausal women. Boxes represent the interquartile range (IQR), central bar the median value, whiskers 1.5-times the IQR, and circles the outlier values. In the un-weighted UniFrac comparison (left panel), the first principal coordinate (PCOA1) accounted for 23% of the variance. In the weighted comparison (right panel), PCOA1 accounted for 52% of the variance.

Forty-eight of the 54 postmenopausal women were included in the microbiota analyses. Fecal microbiota alpha diversity and richness did not differ between women with high versus low mammographic density (Table 3 and S2 Table.). In Fig 2, we assessed if the relative abundance of the microbiota differed at the phylum level, but no statistically significant differences were observed between the 11 phyla by mammographic density status (S2 Table). Furthermore, we completed exploratory analyses of the microbiota at the genus level to assess possible associations with mammographic density, however the few associations were deemed to be an artifact of multiple testing and were determined to be chance findings (Fig 3 and S2 Table). Mammographic density status also was not associated with fecal microbiota beta diversity, either by univariate principal coordinate analysis (Fig 4) or by MiRKAT regression analysis adjusted for BMI, age and current alcohol use (weighted UniFrac P = 0.86, un-weighted UniFrac P = 0.99, data not shown). Although BMI was significantly lower in high-density versus low-density women (P = 0.01, Table 1), gut microbiota composition did not differ between BMI groups (S1 Fig).

**Table 3:** Gut microbiota differences by mammographic density category in 48 postmenopausal women.

Alpha diversity metric	*Low Density (n = 18)*	*High Density (n = 30)*	P-value*
Mean PD tree	38.8 (1.75)	37.8(0.95)	0.67
Mean Shannon	6.3 (0.15)	6.3 (0.09)	0.37
Mean Chao1	1107.6 (51.09)	1067.6 (26.65)	0.28
Mean observed species	92.2 (4.7)	91.8 (2.65)	0.81

* Multiple logistic regression models were adjusted for continuous age, continuous BMI, and current alcohol use. Wilcoxon rank sum analysis of microbiome metrics between low and high density women were also null (P >0.28).

## Discussion

Risk for postmenopausal breast cancer is substantially increased with high mammographic density, and also consistently increased with high estrogen level. Because estrogen is a mitogen that increases mammary epithelial and stromal cell growth [38], higher levels might mediate higher breast density. In contrast to this hypothesis, we observed that postmenopausal women with high mammographic density had significantly lower levels of estrogens and estrogen metabolites (EM) compared to women with low mammographic density. This inverse association between estrogen level and mammographic density in postmenopausal women was also found in several prior studies (S3 Table) [6–9,13–15,17] although it was greatly attenuated with adjustment for BMI, which may be an important confounding variable. In our study, the inverse association between estrogen level and mammographic density persisted after adjustment for BMI (Table 2). Moreover, the effect of BMI on estrogens appeared to be limited to a few obese women with low breast density (Fig 1). Based on limited power, the interaction of BMI and estrogen level on density was not statistically significant (P = 0.77).

Further to the consideration of BMI, in a study of 194 postmenopausal women, Fuhrman, et al. reported that density was 15% higher per decile of estrone in 67 obese women, whereas density was 3.5% lower per decile of estrone in 127 non-obese women (interaction P = 0.02) [17]. Fuhrman, et al. also reported that density was directly related to parent estrogen/EM ratio, but this density association with parent/EM ratio was driven by estrone and estradiol and was only found in the obese women (interaction P = 0.02). We found that density was inversely related to all EM levels, as well as to estrone and estradiol levels, and density in our study was unrelated to parent/EM ratio. One possible explanation for the inverse association between estrogen levels and mammographic density observed in our study is that density and endogenous estrogens may influence breast tissue pathology, and subsequent cancer risk, through completely independent pathways [1]It must be noted that other studies have reported conflicting results, including no association of mammographic density with estrogens in a large European population [10,11,16,18–20], and positive associations in others (S3 Table).

It is well established that the gut microbiota and its products impact human health and disease. Most notably the gut microbiota has been associated with diabetes, obesity, and colon cancer [23]. In addition, the gut microbiota could function as a metabolic incubator and thereby affect breast cancer risk [39,40]. Adlercreutz et al found that treating women with ampicillin increased fecal estrogens, suggesting that the abrupt alteration of the intestinal microbiota affects estrogen levels [41]. Lombardi et al. observed that the microbiota present in feces could metabolize both parent estrogens and estrogen metabolites [42]. Previously we reported that high ratios of estrogen metabolites to parent estrogens were associated with a more diverse gut microbiota in healthy postmenopausal women [29]. These findings complemented our recent case-control study that found less gut microbial diversity among postmenopausal breast cancer cases when compared to controls [24]. In addition, the breast cancer cases had a different microbial composition than control women [24]. To further understand the observed association between the gut microbiota and postmenopausal breast cancer, the current study assessed whether mammographic density was associated with microbial diversity or composition. To our knowledge this is the first study to assess the relationship of the gut microbiota with mammographic density. Although we found no association between the gut microbiota and mammographic density, the microbiota might still affect breast cancer risk through inflammatory, dietary, nutrient or other pathways.

Our study has several strengths. We used data from the electronic medical record to identify a sample of women who met our stringent inclusion criteria, and confirmed their eligibility and menopausal status prior to enrollment. We measured both the fecal microbiota and urinary estrogens using state-of-the-art tools. The microbiota was characterized using pristine, immediately stabilized and frozen feces and next-generation sequencing methods; and a highly sensitive LC-MS/MS assay system was used to detect and accurately quantify estrone, estradiol, and 13 estrogen metabolites [43].

The limitations of our study must be noted. Our sample size was small. With only 54 women for estrogen comparisons and only 48 for microbiota comparisons, our power to detect associations with mammographic density and the gut microbiome was limited. Furthermore, small sample size may have limited our ability to find an interaction effect of BMI on the relationship between mammographic density and estrogen levels. In our cross-sectional design, we cannot distinguish the sequential relationship between mammographic density and urinary estrogen level, although both of these are relatively stable in women who, like ours, are 10 years past menopause [44–49]. Also, we were unable to determine a cause and effect relationship between BMI, estrogen levels, and mammographic density due to lack of temporality in our study. We did not examine possible associations of mammographic density with levels of prolactin, insulin-like growth factor, and other potential mitogens that have been noted in prior studies [6,18,21].

Our use of the clinically determined BI-RADS 5^th^ edition categories to define mammographic density could be considered a limitation, as most prior studies have assessed breast cancer risk based on complex computer-assisted algorithms for characterization and quantification of density [4,16,50–54]. However, BI-RADS 5^th^ edition classification is also a strength, as it is the current standard of care and widely used in clinical medicine and thus will facilitate replication of our findings. Although our BI-RADS categorization was determined by only one radiologist, with no independent validation, agreement was very high in an independent study in which 1000 cases were read by five radiologists weighted kappa 0.88 [36]. The error inherent in a single reading and the lower precision with BI-RADS categories than with a computer-assisted scale would reduce statistical power to detect true associations.

Despite our study’s limitations, we did detect a strong, statistically significant association between high mammographic density and low endogenous estrogen levels in postmenopausal women. This association, which was clearly independent of BMI and which reflected not only estrone and estradiol but also their metabolites, requires independent validation in varied populations, ideally with finer resolution of mammographic density and prospective follow-up to establish sequential relationships. An observed inverse association could indicate independent breast cancer risk pathways via systemic estrogens and mammographic density. Future studies will be required to clarify the independent or, more likely, joint effects of high density and high estrogens on breast cancer risk. Ultimately, large prospective cohort studies will be needed to examine the independent and joint effects of estrogen levels, mammographic density, gut microbiota metrics, and traditional variables on breast cancer risk.

## Supporting information

S1 File Breast and colon health study questionnaire. (PDF)

S1 Table Estrogen and estrogen metabolite (EM) associations with mammographic density by original BI-RADS classification. (DOC)

S2 Table Taxonomic associations with mammographic density among postmenopausal women. (XLSX)

S3 Table Literature review of publications that assessed the association between mammographic density (MD) and parent estrogens and estrogens metabolites in healthy postmenopausal women. (DOC)

S1 Fig Box plot comparisons of beta diversity by body mass index (BMI) category. (TIF)
