# Supplementary material for: Mammographic breast density and its association with urinary estrogens and the fecal microbiota in postmenopausal women
Source: PLoS One. 2019 May 8;14(5):e0216114. doi: 10.1371/journal.pone.0216114 (PMC6505928; doi:10.1371/journal.pone.0216114)
Supplement: S1 File — (PDF) [file pone.0216114.s001.pdf]

Breast and Colon Health (BRanCH) Study Lifestyle Survey

Instructions:

1. Thank you for participating in the BRanCH study. We are grateful for your commitment to this important research. This survey should take 20 minutes to complete. Please make an effort to complete every question. If unsure, estimate to the best of your ability. Place an "X" in the box next to your answer and write in your response where necessary.
2. The last 2 pages of this form must be completed AFTER you collect the samples. For ease, you may choose to complete the entire form after collecting the samples.
3. Please mail the completed form in the postage-paid, pre-addressed envelope provided. DO NOT INCLUDE THIS COMPLETED FORM WITH THE SAMPLES.

Completing this survey is voluntary. If you have questions or DO NOT want to participate in the survey or study, please call the BRanCH study staff, at 303-614-1201. Some questions may be sensitive, and you may skip any question you do not want to answer. To protect your confidentiality, your survey will only be identified with a subject ID number.

By completing this survey, you are consenting to participate in our study. In addition to the information and specimens you are providing, the study will also access information in your medical record, which may include history of mammograms, breast biopsies, or medication use.

The Basics

1. Today's date

Month			Date			Year			
		/			/				

2. Are you of Spanish origin, Hispanic or Latino?

	YES		NO

3. Race (please check all that apply)

	Native American or Alaska Native
	Asian
	Black or African American
	Native Hawaiian or Other Pacific Islander
	White
	Other (please specify: ______________________________)

4. What is the highest level of schooling that you have completed?

	Some high school or less
	High school graduate or GED
	Some college or technical school
	Graduated college
	Postgraduate degree

SUBJECT ID: ___________

Menstrual and Reproductive History

5. How old were you when you started having your menstrual period?

		Age

6. How old were you when you stopped having your menstrual period?

		Age

7. Which of the following best describes why your periods stopped? (Please select only one answer.)

	Natural menopause (change of life)
	Surgery (uterus and/or ovaries surgically removed)
	Endometrial ablation
	Other (please specify): ____

9. Have you ever had surgery on a reproductive organ?

	YES
	NO (Skip to 11)

10. Which operation(s) did you have and in what year(s): (Mark all that apply)

		YEAR OF SURGERY
	Uterus removed (hysterectomy)	
	One ovary removed (oophorectomy)	
	Both ovaries removed (bilateral oophorectomy)	
	Ovaries may have been removed	
	Other, please specify: ____________________	

11. How many times have you been pregnant (include miscarriage, ectopic pregnancy, etc.)?

		# Pregnancies
	Never (Skip to 19)	

12. Have you ever had a live birth?

	YES
	NO (Skip to 19)

13. How many live births have you had (include term and pre-term)?

		Total deliveries

15. At what age did you have your first live birth?

		Age

SUBJECT ID: ___________

16. At what age did you have your last live birth?

		Age
	One live birth only	

14. Have you delivered twins or other multiples?

	YES		NO

17. Did you breastfeed any of your babies?

	YES		NO (Skip to 19)

18. How many months did you breastfeed, in total, including all your babies?
(Add up the total months for all your babies. For example, if you breastfed 2 babies for one year each, you would report 24 months.)

		Total months

19. Have you EVER USED prescription hormones (HRT) for relief of menopausal symptoms, irregular periods, or prevention of disease such as bone loss?

	YES
	NO (Skip to 21)

20. How old were you when you STOPPED using prescription hormones?

		Age

21. Have you EVER used Raloxifene?

	YES		NO

22. Have you EVER used Tamoxifen?

	YES		NO

23. Are you currently using any over-the-counter products for the relief of menopausal symptoms (including any hormone creams)? If yes, please provide the name of product.

	YES, product name: ______________________________
	NO

Breast Health

24. Have you ever had a breast biopsy?

	YES
	NO (Skip to 28)

25. How many breast biopsies have you had?

		# Biopsies

SUBJECT ID: ___________

26. At what age was your MOST RECENT breast biopsy? ☐☐ Age

27. Have you ever been diagnosed with atypical hyperplasia (from a breast biopsy)? ☐ YES ☐ NO ☐ Don't Know

Lifestyle Factors

28. Have you smoked at least 100 cigarettes in your lifetime?

☐	NO (Skip to 31)
☐	Yes, and I currently smoke
☐	Yes, but I no longer smoke

29. How many total years have you smoked? (Please subtract time when you did not smoke.)

☐☐ Years

30. How many cigarettes do/did you smoke per day on average?

☐☐ Cigarettes per day

31. Do you currently drink alcoholic beverages (beer, wine or liquor)?

☐	YES
☐	NO but I have in the past (Skip to 33)
☐	NO, never drank alcohol (Skip to 33)

32. How many drinks do you usually have (one drink equals 12 ounce bottle/can of beer, a shot glass of liquor, or 5 ounces of wine)?

☐	Less than 1 drink/month
☐	1-4 drinks per MONTH
☐	1-6 drinks per WEEK
☐	1-2 drinks per DAY
☐	3 or more drinks per DAY

33. Now think about your physical activity over the last twelve months, ignoring any recent changes. How often did you participate in vigorous physical activities such as lap swimming, jogging/running, fast cycling or on hills, aerobics, downhill or cross-country skiing, or elliptical or other aerobic machine?

☐	Never
☐	<1 hour per week
☐	1-<2 hours per week
☐	2-<3 hours per week
☐	3-<5 hours per week
☐	5 or more hours per week

SUBJECT ID: ___________

34. Over the past twelve months, how often did you participate in moderate activities such as brisk walking, recreational tennis, leisurely cycling on level streets, golfing (without a cart), yoga/pilates, or ballroom dancing?

	Never
	<1 hour per week
	1-<2 hours per week
	2-<3 hours per week
	3-<5 hours per week
	5 or more hours per week

35. Currently, how many hours per week do you typically spend gardening, working in the yard, shopping, performing home maintenance or housekeeping?

	Never
	<1 hour per week
	1-<2 hours per week
	2-<3 hours per week
	3-<5 hours per week
	5 or more hours per week

36. What is your current height and weight?

Current Height:		ft		in	Current Weight:				pounds

37. How much did you weigh when you were 20 (if you were pregnant at age 20, estimate your pre-pregnancy weight)?

			Weight at age 20

38. When you gain weight, where on your body do you mainly tend to add the weight (mark all that apply)?

	Don't Gain Weight
	Around the waist / stomach
	Around the hips and thighs
	Equally all over
	Other (please specify): ______________________________

Family History of Breast Cancer

39. How many biological daughters do you have?

	# of biological daughters
	NONE (Skip to 41)

40. How many of these daughters have had breast cancer?

	# of biological daughters with breast cancer
	NONE

SUBJECT ID: __________

41. Are you adopted?

	YES (Skip to 45)
	NO

42. Did your biological mother have breast cancer? ☐ YES ☐ NO ☐ Don't know

43. How many biological ("full") sisters do you have?

	# of biological sisters
	NONE (Skip to 45)

44. How many of these sisters have had breast cancer?

	# of full sisters with breast cancer
	NONE

Diet and Colon Health

These questions are about conditions that may affect the stool sample you are providing.

45. How often do you usually have a bowel movement?

	More than once per day
	Once per day
	Every other day
	Every few days
	Less frequently

46. Are your stools loose or watery?

	Usually
	Sometimes
	Rarely

46. Are your stools hard or difficult to pass?

	Usually
	Sometimes
	Rarely

47. Have you taken any antibiotics by mouth or injection in the past two weeks?

	YES, by mouth
	YES, injection
	NO (Skip to 50)

48. What date did you START the antibiotic?	Month:____-____<br>Date: ____-____	
49. What date did you STOP the antibiotic?	Month:____-____<br>Date: ____-____ OR:	
		CURRENTLY USING ANTIBIOTIC

SUBJECT ID: ___________

50. In the past year, did a doctor ask you to collect a stool sample to check for infection?

☐ YES ☐ NO

51. Have you ever been diagnosed with Celiac Disease? ☐ YES ☐ NO

52. Please indicate if you have any food allergies or adhere to any dietary restrictions (mark all that apply)?

	NONE
	Vegan
	Vegetarian (NO fish, chicken or meat)
	No wheat (or gluten)
	No or limited milk (or lactose)
	No tree nuts or peanuts
	Other (Kosher, etc) LIST: ______________________________

53. Do you regularly (at least once per week) consume any probiotic tablet, yogurt, drink or other product? (List all that apply)

	YES, product name: ______________________
	YES, product name: ______________________
	YES, product name: ______________________
	NO

54. Do you regularly (at least once per week) consume any fiber supplement?

	YES, product name: ______________________
	NO

55. Do you regularly (at least once per week) consume any soy supplement?

	YES, product name: ______________________
	NO

SUBJECT ID: ___________

STOP! Please complete the rest of these questions AFTER you have given your stool and urine samples. Thank you!!

56. We're interested in knowing the last time you ate the foods listed below. Only mark <u>one answer</u> for each food item.

Mark only ONE answer for each food item.	Ate within last 24 hours	Ate 1-3 days ago	Ate 4-7 days ago	Did not eat in the last 7 days or never eat
PROCESSED OR CURED MEATS/POULTRY				
Ham (as a main dish or lunch meat)	☐	☐	☐	☐
Bacon (pork or turkey)	☐	☐	☐	☐
Beef or pork sausage (links or patties)	☐	☐	☐	☐
Chicken or turkey sausage (links or patties)	☐	☐	☐	☐
Beef or pork cold cuts (e.g., bologna, pepperoni, salami, pastrami, corned beef, prosciutto, etc.)	☐	☐	☐	☐
Chicken or turkey cold cuts (including turkey bologna)	☐	☐	☐	☐
Beef hotdogs or frankfurters	☐	☐	☐	☐
Chicken or turkey hotdogs or frankfurters	☐	☐	☐	☐
FRESH MEATS/POULTRY (NOT PROCESSED)				
Beef (e.g., steaks, hamburgers, beef stew)	☐	☐	☐	☐
Pork (e.g., roast, chops, ribs)	☐	☐	☐	☐
Chicken or turkey (including ground meat)	☐	☐	☐	☐
VEGETABLES				
Broccoli, cauliflower, or cabbage	☐	☐	☐	☐
Lettuce (all types)	☐	☐	☐	☐
Spinach, or other dark green leafy vegetables like kale, collard, or mustard greens	☐	☐	☐	☐
Celery	☐	☐	☐	☐
Beets, radishes or rhubarb	☐	☐	☐	☐

SUBJECT ID: ___________

Feasibility Questions

These questions will help us improve the study. Please complete this section AFTER you have collected the urine and stool samples and completed the online questionnaire. Please add any specific comments you have for the study team. We appreciate the input and ideas!

57. Did you find the directions for the stool and urine collection clear and easy to follow?

	YES
	NO<br>Please tell us what could have been clearer:

58. Was collecting the stool sample more difficult or unpleasant than you expected?

	NO
	YES<br>Please tell us why:

59. Did you have any problems packaging or shipping the samples?

	NO
	YES<br>Please tell us your problem:

60. Did you complete the online diet questionnaire at https://www.nutritionquest.com/login/?

	YES (Skip to 62)
	NOT YET but will complete (Skip to 62)
	WILL NOT COMPLETE

61. Please tell us why you will not complete the online diet questionnaire:

	No computer or internet access
	Problems with computer or logging on to site
	Other:

62. Please provide any other comments or suggestions to the study team:

____

____

Thank you for taking the time to complete this questionnaire. Please return it in the pre-addressed postage-paid envelope provided.

SUBJECT ID: __________
