# Supplementary material for: Mammographic breast density and its association with urinary estrogens and the fecal microbiota in postmenopausal women
Source: PLoS One. 2019 May 8;14(5):e0216114. doi: 10.1371/journal.pone.0216114 (PMC6505928; doi:10.1371/journal.pone.0216114)
Supplement: S1 Table — (DOC) [file pone.0216114.s002.doc]

| **Supplementary Table 1. Estrogen and estrogen metabolite (EM) associations with mammographic density by original BI-RADS classification.** | | | | | |
| --- | --- | --- | --- | --- | --- |
|  | **Almost Entirely Fatty (N=6)** | **Scattered Fibroglandular (N= 17)** | **Heterogeneously Dense (N= 26)** | **Extremely Dense (N=5)** | **P-value*** |
| **Estrogen, EM levels (SD)** |  |  |  |  |  |
| **Mean Total Estrogen** | 22.5 (4.8) | 29.4 (24.4) | 18.3 (11.5) | 19.9 (19.9) | 0.02 |
| **E1** | 6.6 ( 2.1) | 7.4 (6.6) | 4.9 ( 3.4) | 4.8 (4.8) | 0.02 |
| **E2** | 1.6 (0.57) | 1.8 (1.8) | 1.1 (0.64) | 1.5 (1.4) | 0.05 |
| **Parent Estrogens** | 8.3 (2.3) | 9.2 (8.4) | 6.0 (3.8) | 6.3 (6.2) | 0.02 |
| **Estrogen Metabolites** | 14.2 ( 4.3) | 20.1 (16.8) | 12.3 (8.1) | 13.5 (13.5) | 0.03 |
| **16-Pathway** | 7.4 (2.4) | 10.9 (9.4) | 6.4 (4.3) | 6.8 (6.6) | 0.02 |
| **2- Pathway** | 6.2 (1.9) | 8.4 (7.6) | 5.4 (3.5) | 6.1 (6.3) | 0.04 |
| **4-Pathway** | 0.5 (0.1) | 0.7 (0.3) | 0.5 (0.3) | 0.6 (0.6) | 0.05 |
|  |  |  |  |  |  |
| **EM/parent ratio** | 1.82 (0.65) | 2.82 (3.22) | 2.10 (0.66) | 2.51 (1.27) | 0.80 |
| **16-Pathway /parent ratio** | 0.9 (0.4) | 1.7 (2.6) | 1.1 (0.4) | 1.2 (0.7) | 0.77 |
| **2- Pathway /parent ratio** | 0.8 (0.3) | 1.0 (0.6) | 0.9 (0.3) | 1.2 (0.6) | 0.76 |
| **4-Pathway /parent ratio** | 0.07 (0.02) | 0.09 (0.06) | 0.08 (0.03) | 0.11 (0.06) | 0.63 |
| **2-Pathway /16-pathway ratio** | 0.85 (0.13) | 0.84 (0.21) | 0.85 (0.12) | 0.98 (0.30) | 0.19 |

* Linear regression models adjusted for age and BMI.
