# Supplementary material for: Mammographic breast density and its association with urinary estrogens and the fecal microbiota in postmenopausal women
Source: PLoS One. 2019 May 8;14(5):e0216114. doi: 10.1371/journal.pone.0216114 (PMC6505928; doi:10.1371/journal.pone.0216114)
Supplement: S3 Table — (DOC) [file pone.0216114.s004.doc]

| **Supplementary Table 3. Literature review of publications that assessed the association between mammographic density (MD) and parent estrogens and estrogens metabolites in healthy postmenopausal women** | | | | | |
| --- | --- | --- | --- | --- | --- |
| **Author (Year)** | **N** | **Biospecimen** | **Estrogen Measurement Method** | **MD Assessment Method** | **Association** |
| Aiello et al.(2005) | 88 | Plasma | Radioimmunoassay (RIA) | Cumulus 108 | Inverse (estradiol, estrone, free estradiol) |
| Boyd et al. (2002) | 189 | Serum | Radioimmunoassay (RIA) | Radiologist - computer assisted | Inverse ( free estradiol) |
| Bremnes et al (2007) | 722 | Plasma | Radioimmunoassay (RIA) | Radiologist - computer assisted | Positive ( estrone ) |
| Fuhrman et al. (2012) | 194 | Urine | Liquid chromatography, tandem mass spectrometry (LC - MS/MS) | Radiologist - computer assisted | Inverse ( estrogen metabolites and estrogen ratios), positive for parent estrogen/estrogen metabolite ratio) |
| Gierach et al. (2015) | 87 | Serum | Liquid chromatography, tandem mass spectrometry (LC - MS/MS) | SXA Phantom | Positive ( estrone, estradiol, estrogen metabolites), Inverse (estrogen metabolite ratios)* |
| Greendale et al (2005 ) | 365 | Serum | Radioimmunoassay (RIA) | Radiologist - computer assisted | Positive (estrone, estradiol, bioavailable estradiol) |
| Johansson et al. (2008) | 174 | Serum | Radioimmunoassay (RIA) | Radiologist - computer assisted | Positive ( estradiol) |
| Jones et al. (current study) | 54 | Urine | Liquid chromatography, tandem mass spectrometry (LC - MS/MS) | BI-RADS, dichotomized | Inverse (estrone, estradiol, estrogen metabolites) |
| McCormack et al. (2009) | 270 | Plasma | Radioimmunoassay (RIA) | Cumulus | Inverse (estradiol, estrone free estradiol)* |
| Riza et al. (2001) | 140 | Urine | Enzyme immunoasay kit | Radiologist- Wolfe assessment | Positive ( 2- hydroxestrone, 16 alpha- hysroxyestrone, ratio of 2-hydroxyestrone : 16 alpha- hydroxyestrone) |
| Schoemaker et al. (2014) | 334 | Plasma | Radioimmunoassay (RIA) | Cumulus | Inverse (estrone, estradiol, free estradiol)* |
| Sprague et al. (2011) | 257 | Serum | Radioimmunoassay (RIA) | Cumulus | Inverse ( estradiol, estrone, free estradiol, bioavailable estradiol), No association (estrone sulfate )* |
| Tamimi et al. (2005) | 485-515 | Plasma | Radioimmunoassay (RIA) | Cumulus | Inverse ( estrone estradiol, free estradiol, estrone sulfate)* |
| Verheus et al. (2007) | 775 | Plasma | Radioimmunoassay (RIA) | Radiologist - computer assisted | Inverse (estradiol, estrone free estradiol)* |
| Warren et al. (2006) | 1413 | Plasma | Radioimmunoassay (RIA) | Radiologist- Wolfe/Boyd Assessment | No association ( estrone estradiol, free estradiol, estrone sulfate)* |
| Woolcot et al. (2013) | 297 | Serum | Radioimmunoassay (RIA) | Radiologist- computer assisted | Positive (estradiol)* |

* No Association when adjusted for BMI.
