# Supplementary figures and images for: Mammographic breast density and its association with urinary estrogens and the fecal microbiota in postmenopausal women
Source: PLoS One. 2019 May 8;14(5):e0216114. doi: 10.1371/journal.pone.0216114 (PMC6505928; doi:10.1371/journal.pone.0216114)

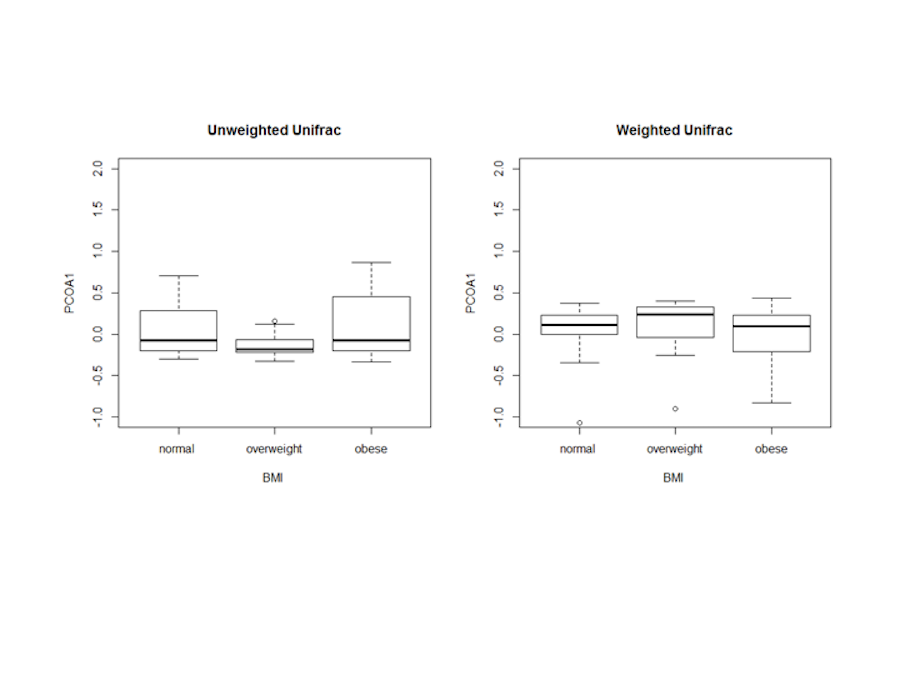

Supplement: S1 Fig — (TIF) [file pone.0216114.s005.tif]
